# International trends in pulmonary neuroendocrine cancer studies:a scientometric study

**DOI:** 10.31744/einstein_journal/2022RW0113

**Published:** 2022-10-13

**Authors:** Hugo Tanaka, Auro del Giglio

**Affiliations:** 1 Centro Universitário FMABC Santo André SP Brazil Centro Universitário FMABC, Santo André, SP, Brazil.

**Keywords:** Scienciometry, Scientometric analysis, Lung neoplasms, Neuroendocrine tumors

## Abstract

**Introduction:**

Pulmonary neuroendocrine tumors account for approximately 20% of all primary lung tumors. Few studies summarize the current body of pulmonary neuroendocrine tumors studies worldwide.

**Objective:**

A quantitative scientometric analysis was conducted to evaluate the development of applications and innovations and to analyze their contribution to various areas of improvement in treatment and diagnosis of pulmonary neuroendocrine tumors.

**Methods:**

We searched for studies published in the last 20 years in the databases United States National Library of Medicine (PubMed), Scientific Electronic Library Online (SciELO), Scopus, and Web of Science, using the terms ‘pulmonary neuroendocrine tumors’, ‘bronchial neuroendocrine tumors’, ‘bronchial carcinoid tumors’, ‘pulmonary carcinoid’, ‘typical pulmonary carcinoid’, ‘atypical pulmonary carcinoid’, ‘pulmonary carcinoid and diagnosis’, ‘pulmonary carcinoid and treatment’, ‘pulmonary carcinoid and epidemiology’ and ‘pulmonary carcinoid and prognosis’.

**Results:**

Our results showed the number of publications increased significantly over the study period and was strongly associated with the economic or financial situation of the publications’ countries of origin. We observed a predominance of studies on histological diagnosis compared to treatment, and among the studies related to treatment, a predominance of retrospective studies relative to prospective studies was found.

**Conclusion:**

Based on the published literature, we concluded research on pulmonary neuroendocrine tumors still seems to be incipient, because it favors studies related to histological characterization of the disease, and therapeutic studies are still predominantly of a retrospective nature.

## INTRODUCTION

Neuroendocrine neoplasms (NENs) comprise a rare and heterogeneous group of neoplastic diseases that most commonly occur in the gastroenteropancreatic tract (GEP) and lungs.^(1)^ This group is subdivided into ‘neuroendocrine tumors’ (NETs), which are well-differentiated tumors, and ‘neuroendocrine carcinomas’ (NECs), which are poorly differentiated.^(2)^ Pulmonary neuroendocrine tumors (NETp), a type of NEN, account for approximately 20% of all primary lung tumors. These tumors share certain morphological, ultrastructural, immunohistochemical and molecular characteristics but have important differences in incidence and survival, as well as clinical, epidemiological, histological and molecular aspects.^(3,4)^

Carcinoid tumors account for 1% to 2% of invasive lung neoplasms, and only 10% of carcinoids are atypical carcinoids (AC).^(4)^ However, the diagnosis of NETp can be challenging given their morphological similarities with other tumors.^(3)^ There are limited data on the results of systemic treatment for NETp with different cell morphologies.^(2,3)^

Among the types of NETp, typical carcinoids (TC) usually present a favorable prognosis.^(5,6)^ Treatment options for unresectable/metastatic TC include somatostatin analogs and everolimus; to date, there is no consensus on the use of chemotherapy for TC patients.^(3,7)^ In contrast, AC seem to be more common, with a higher rate of distant and nodal metastases and a lower five-year survival rate, even when metastatic disease is present.^(8-10)^

There is no proven optimal therapy for unresectable metastatic TC or AC,^(4)^ and it is also noteworthy that the epidemiology, clinical behavior and treatment of TC and AC differ significantly from those of other lung neoplasms, and clinical data are limited due to the small number of patients with these cancers.^(4,11)^ Few studies summarize the current state of the art of global research on NETp. In a literature review, Tsoukalas et al.^(12)^ noted advances in systemic treatment for pulmonary NENs and summarize updated information on the treatments used. However, at present, no available studies have evaluated how global research groups address the subject, what aspects of the topic are most studied and which countries have made pioneering and promising advances in NETp research.

Scientometric studies addressing NENs as well as NETp are of significant importance for understanding the evolution of this area of medicine, and they contribute to the periodic ordering of available information and results, which leads to innovation. With such studies, it is possible to articulate and integrate different perspectives that previously stood alone and to identify inconsistencies and gaps. Therefore, this study presents the worldwide scientific production regarding these issues through a scientometric analysis of the publications in the area of NETp over the last 20 years.

## METHODS

### Literature search

Literature searches were performed at the United States National Library of Medicine (PubMed), Scientific Electronic Library Online (SciELO), Scopus, and Web of Science, using the terms ‘pulmonary neuroendocrine tumors’, ‘bronchial neuroendocrine tumors’, ‘bronchial carcinoid tumors’, ‘pulmonary carcinoid’, ‘typical pulmonary carcinoid’, ‘atypical pulmonary carcinoid’, ‘pulmonary carcinoid and diagnostic’, ‘pulmonary carcinoid and treatment’, ‘pulmonary carcinoid and epidemiology’ and ‘pulmonary carcinoid and prognosis’. The Boolean operators “AND” and “OR” were used to associate the keywords when necessary.

Publications marked “articles” were taken into account. The list of publications was limited to keywords defining the field of research, thus allowing to filter out most publications by other branches of oncology that were not related to the specific area of pulmonary neuroendocrine tumors. Each term was first searched without filters; then, filters were applied to search by publication type and consider only studies that presented NETp as the main study object. Bibliographic studies and case reports were not considered. No language restrictions were applied.

The study period was between 2000 and 2020, with the aim of determining the state of the art over the last 20 years.

The following information was collected from the selected publications: year of publication; publication journal; type of study (retrospective, prospective); keywords; countries where the studies were conducted; type of pulmonary neuroendocrine cancer studied and objective/hypothesis of the study.

### Data analysis

The absolute frequency of each qualitative variable was calculated. Quantitative variables were assessed for normality based on histograms and the Shapiro-Wilk test. To compare qualitative variables, the χ^2^test was used, and for quantitative variables, we employed Mann-Whitney test. The correlation test was used to assess the dependence of the number of published articles on the year of publication. All statistical tests were two-sided, with the α level set at 0.05.

## RESULTS

A total of 216 publications were selected from 103 national and international journals. The studies were conducted in 35 countries; eight of the studies were multicenter studies, and 208 were conducted at a single center using clinical data from the research institution or a database.

**Figure 1:**
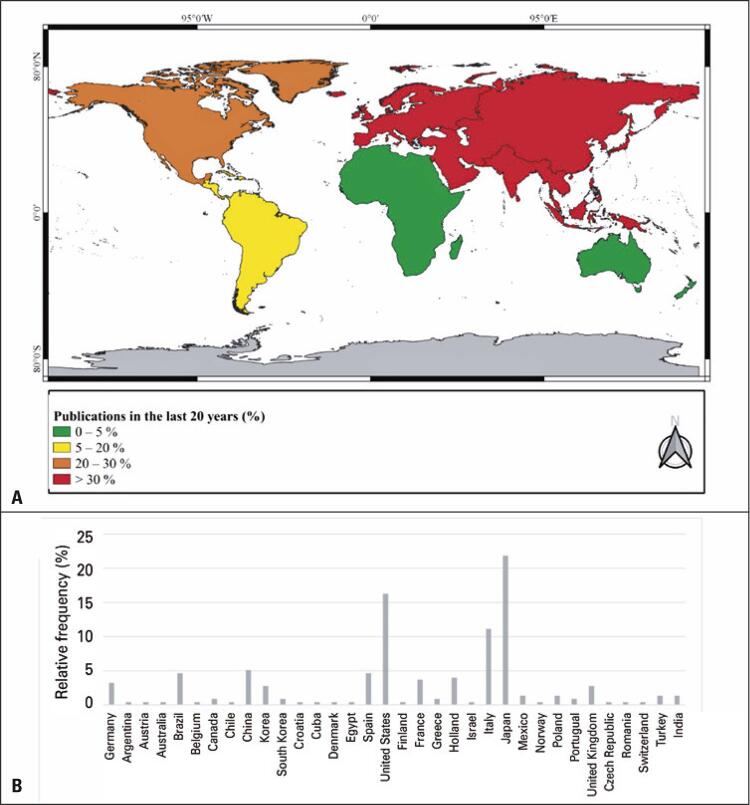
Global distribution of pulmonary neuroendocrine tumors studies. (A) Relative frequency of publications focusing on pulmonary neuroendocrine tumors by continent; (B) Relative frequency of publications focusing on pulmonary neuroendocrine tumors

The articles on pulmonary neuroendocrine tumors were written by researchers in 35 countries; 57.1% of researchers were from Europe, 17.1% from Asia, and 8.6% from North America (Figure 1A). The countries with the highest number of published studies were Japan, with 47 articles (21.9%); the United States, with 25 articles (16.3%); and Italy, with 24 articles (11.1%); together, these countries accounted for almost half of the publications surveyed in this study (Figure 1B).

**Figure 2:**
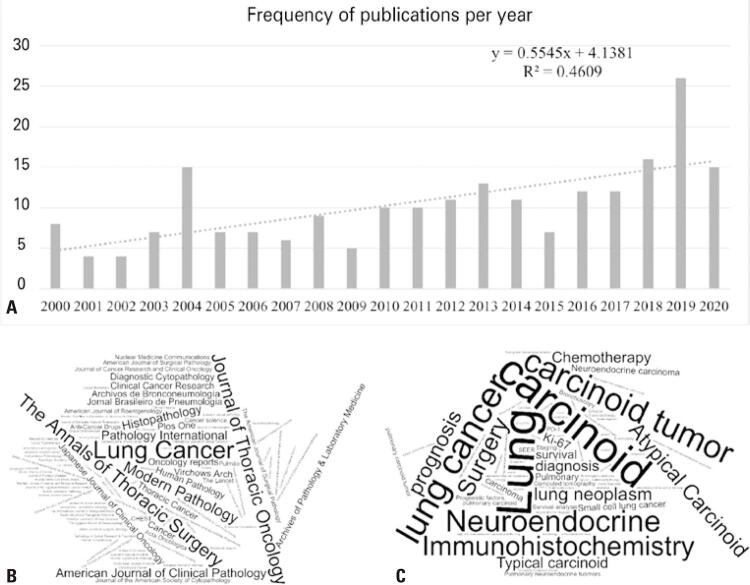
Profile of studies published on pulmonary neuroendocrine tumors in recent years. (A) Relative frequency of published articles focusing on pulmonary neuroendocrine tumors by year of publication; (B) Word cloud of journals by the relative frequency of the number of publications focusing on pulmonary neuroendocrine tumors; (C) Word cloud of the relative frequency the keywords in articles focusing on pulmonary neuroendocrine tumors

A temporal trend was observed (Figure 2A), and there was a positive correlation between the number of articles published and the year of publication (R^2^=0.46; F=16.241; p=0.001). Of the 103 journals represented in this study, those that published the most articles focusing on NETp were Lung Cancer, European Journal of Cardiothoracic Surgery, The Annals of Thoracic Surgery and Journal of Thoracic Oncology (17, 14, 13 and 10 articles, respectively); together, these journals accounted for 25% of publications in the last 20 years (Figure 2B). In addition, the average number of articles published as a function of the year of publication presented a positive correlation; this shows that, in general, journals increased the number of articles published on this topic in the last 20 years (R^2^=0.55; F=1.346; p=0.05).

Regarding the nature of the studies, 25 were prospective, and 191 were retrospective, including evaluations of medical records or database information or reassessment of biopsy samples. It is noteworthy that case studies were excluded from the search because they did not characterize the neoplasia but only described the specific case. The most frequently used keywords (Figure 2C) in the selected articles were ‘large cell neuroendocrine carcinoma (LCNEC)’ (27.91%), ‘lung’ (19.19%), ‘carcinoid’ (16.86%), ‘neuroendocrine tumors’ (16.28%), ‘lung cancer’ (15.70%).

**Figure 3:**
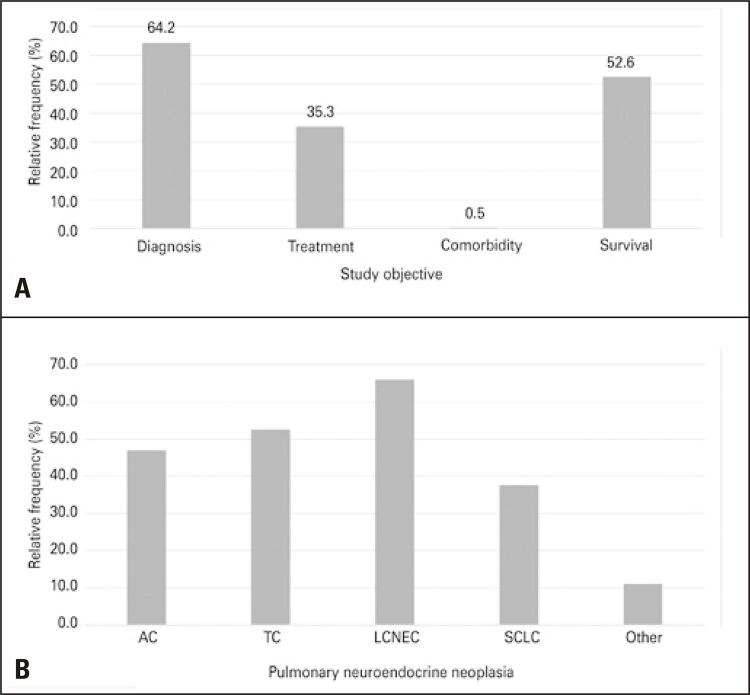
Themes discussed in the papers published in recent years. (A) Relative frequency of publications focusing on pulmonary neuroendocrine tumors by the objectives addressed; (B) Relative frequency of publications focusing on pulmonary neuroendocrine tumors as a function of the type of pulmonary neuroendocrine neoplasia

AC: atypical carcinoids; TC: typical carcinoids; LCNEC: large cell neuroendocrine carcinoma; SCLC: small cell neuroendocrine carcinoma.

The main objectives of the studies were related to tumor differentiation and treatment. Of the 216 studies, 64.19% assessed the techniques for diagnosing these lung cancers by evaluating their morphology, ultrastructure, immunohistochemistry, and molecular characteristics (Figure 3A). A total of 35.35% of studies aimed to evaluate and/or compare treatments. Survival was most often evaluated in association with other objectives, and 52.56% of studies evaluated different forms of survival among patients to complement their results (Figure 3A).

Regarding the pulmonary NENs addressed in the studies, most investigations considered more than one neoplasia for evaluation, and LCNEC was the most addressed in the last 20 years (considered in 66% of studies), followed by TC (52.6%), AC (47%) and small cell neuroendocrine carcinomas (SCLC). In addition, 11.2% of studies compared one of the four types of NETp with another type of lung cancer (Figure 3B).

## DISCUSSION

No scientometric analysis addressing the state-of-the-art of research on NETp, either national or international, has been published to date, and this study provides relevant knowledge to documentation of the current status of scientific production in this field of medicine. Our study is the first to characterize the worldwide scientific activity regarding NETp; as such, it showed a predominance of retrospective studies and studies aimed at the histological characterization of tumors for diagnostic purposes.

Our results showed the publication trends related to the country of origin and the fields of study addressed in the articles published in recent years that prospectively or retrospectively evaluated NETp patients. The number of publications increased over time, evidently due to greater knowledge of pathology, advances in anatomical and functional images of the disease, and the consequent allocation of resources for research regarding diagnosis and efficacy of neoplasia treatments.^(13)^

It is evident that Japan and the United States lead in terms of the number of publications in the field of health thanks to their greater availability of financial resources and trained professionals. In addition, the European continent, which is dominant given the number of publications produced by research groups from European countries, has been able to publish a greater number of studies with multicenter data, considering the European Society of Thoracic Surgeons has structured the Lung Neuroendocrine Tumors Working Group, which has enabled evaluation of large samples.^(14,15)^

In 2013, countries such as India, China and Brazil spent approximately 4%, 5% and 9%, respectively, of their gross domestic product on health, while Japan, Canada and the United States spent approximately 10%, 11% and 17%, respectively.^(16)^ In a survey of clinical trial records of some types of breast cancer, Ramaswami et al. indicated most clinical trials took place in the United States and in European countries; in contrast, no trials were conducted in African countries.^(17)^ As a result of the scarcity of investment in public health systems, low investment in research and gaps in training and education, access to clinical cancer trials is limited.^(18)^

Thus, it can be said the number of publications may be strongly associated with the economic or financial situation of a given country due to its relation with availability of equipment and medication for diagnosis and treatment, and access to investment and skilled labor for the advancement of research and development.^(19)^ In addition, large funders favor centers that are local to them rather than funding research in peripheral countries;^(20)^ consequently, regions with funders produce more studies and train more qualified professionals in these sectors.

In this survey, we observed a predominance of retrospective studies of diagnostic and clinical nature. In an international survey, Casciano et al. reported the clinical resources most used by patients with NETs and emphasized that almost half of the patients used chemotherapy; in addition, they had high rates of hospitalization, surgery and use of somatostatin analogs,^(21)^ which reflects the most frequently evaluated treatment methods in studies in recent years. However, the same study indicated some variation in the use of resources among countries, showing that targeted therapies were not widely used in all countries; *e.g.,* France had the highest use of resources, which suggests that French physicians have greater acceptance of targeted therapies than physicians in the other countries studied.^(21)^ Additionally, the National Cancer Institute of the United States emphasizes the need for rigorous evaluation of new agents for this disease, which may lead to results that change clinical practice.^(22)^ Thus, the trend of an increasing number of studies that evaluate different lines of treatment for each tumor type should continue until a consensus is reached on the best way to allocate them.

Regarding the objective of the investigations, there was a predominance of studies evaluating diagnostic techniques over those assessing treatment efficacy. This suggests this area of the literature is still incipient in oncology, as diagnostic aspects predominate over therapeutic aspects. Furthermore, among the treatment studies, retrospective studies predominate; this also represents a difficulty, since retrospective studies are limited in terms of real-time monitoring of patient and disease progression.
